# Eating Behavior and Satiety With Virtual Reality Meals Compared With Real Meals: Randomized Crossover Study

**DOI:** 10.2196/44348

**Published:** 2023-08-10

**Authors:** Alkyoni Glympi, Dorothy Odegi, Modjtaba Zandian, Per Södersten, Cecilia Bergh, Billy Langlet

**Affiliations:** 1 Division of Clinical Geriatrics Center for Alzheimer Research, Department of Neurobiology, Care Sciences and Society Karolinska Institutet Huddinge, Stockholm Sweden; 2 Mandometer Clinics Stockholm Sweden

**Keywords:** exposure therapy, eating behavior, anorexia nervosa, bulimia nervosa, binge eating disorder, overweight, obesity, immersive virtual reality, VR, virtual reality

## Abstract

**Background:**

Eating disorders and obesity are serious health problems with poor treatment outcomes and high relapse rates despite well-established treatments. Several studies have suggested that virtual reality technology could enhance the current treatment outcomes and could be used as an adjunctive tool in their treatment.

**Objective:**

This study aims to investigate the differences between eating virtual and real-life meals and test the hypothesis that eating a virtual meal can reduce hunger among healthy women.

**Methods:**

The study included 20 healthy women and used a randomized crossover design. The participants were asked to eat 1 introduction meal, 2 real meals, and 2 virtual meals, all containing real or virtual meatballs and potatoes. The real meals were eaten on a plate that had been placed on a scale that communicated with analytical software on a computer. The virtual meals were eaten in a room where participants were seated on a real chair in front of a real table and fitted with the virtual reality equipment. The eating behavior for both the real and virtual meals was filmed. Hunger was measured before and after the meals using questionnaires.

**Results:**

There was a significant difference in hunger from baseline to after the real meal (mean difference=61.8, *P*<.001) but no significant change in hunger from before to after the virtual meal (mean difference=6.9, *P*=.10). There was no significant difference in food intake between the virtual and real meals (mean difference=36.8, *P*=.07). Meal duration was significantly shorter in the virtual meal (mean difference=–5.4, *P*<.001), which led to a higher eating rate (mean difference=82.9, *P*<.001). Some participants took bites and chewed during the virtual meal, but the number of bites and chews was lower than in the real meal. The meal duration was reduced from the first virtual meal to the second virtual meal, but no significant difference was observed between the 2 real meals.

**Conclusions:**

Eating a virtual meal does not appear to significantly reduce hunger in healthy individuals. Also, this methodology does not significantly result in eating behaviors identical to real-life conditions but does evoke chewing and bite behavior in certain individuals.

**Trial Registration:**

ClinicalTrials.gov NCT05734209, https://clinicaltrials.gov/ct2/show/NCT05734209

## Introduction

Eating disorders and obesity constitute severe health problems in Western society. Globally, the prevalence of eating disorders has increased recently by 25% [1], and for obesity, it is estimated that, by 2030, 20% of the world's adult population will be obese [2]. Both health conditions considerably impair physical health, disrupt psychosocial functioning [1,3], and are risk factors for depression and anxiety disorders [3,4]. Both are expensive disorders, with the overall cost of eating disorders in the United States in 2018 estimated at $64.7 billion per year [5] and of high BMI worldwide in 2017 estimated to be $802 billion [6].

Although both health problems are related to food consumption, their commonalities are seldom discussed. For instance, the same eating behavior parameters (ie, eating rate and food intake) have been identified to increase the risk of developing anorexia and obesity [7,8]. A majority of these individuals have lost the ability to experience satiety during and after meals [7]. A key characteristic of anorexia nervosa is a slow eating rate, which stems from an effort to prevent weight gain by restricting food intake [7]. People with bulimia nervosa try to restrict their food intake to control their body weight but eventually become sufficiently hungry that they consume large amounts of food rapidly before purging [9]. Binge eaters have the bulimia intake pattern without purging [9]. Those who become obese often try to restrict their food intake, but their efforts are typically transitory and ineffective. Finally, a high eating rate is associated with increased food intake [8], excess body weight [8,10], and a larger bite size [8,11].

Eating disorders and obesity constitute severe health problems, and although their prevalence has increased over the years, there is still no consensus for their treatment [1]. The effectiveness of conventional eating disorder treatments, such as family-based therapy, psychopharmacological treatment, and cognitive behavior therapy, are weak [7], with most studies reporting poor long-term treatment outcomes [12]. Conventional treatment for obesity includes lifestyle modification (diet and exercise), medication, and bariatric surgery. The weight loss associated with lifestyle modification and medication interventions is also limited, due to poor adherence rates [13], and although bariatric surgery is the most effective weight loss treatment, it is associated with multiple health complications [14].

High relapse rates are reported for both eating disorders and obesity, with only some people with anorexia nervosa reaching full recovery [15] and people with obesity often regaining most of the lost weight [16]. Last, high dropout rates are another issue that affects the treatment outcomes. For instance, it is estimated that, for people with anorexia nervosa, the dropout rates range from 20.2% to 49.6% [17] and, for people with obesity, from 10% to more than 80%, depending on the treatment program [18]. Therefore, there is an urgent need to explore effective alternative treatments to address these health problems.

Virtual reality devices have been used in many areas of health care for a variety of applications from professional health care education to disease treatment [19]. Examples of conditions that have been treated successfully with virtual reality are anxiety disorders, phobias, stress management, posttraumatic stress disorder, acute and chronic pain management, autism, and physical rehabilitation [20,21]. An especially promising area of use may be for the assessment and treatment of eating disorders and obesity, for which virtual reality devices can be used as an adjunctive treatment tool [20,22,23].

Recent technological advancements have significantly decreased the cost of virtual reality, making the technology more accessible for medical treatments [24]. The ability to customize and control the virtual reality environment makes the application of this technology easier in a treatment context, since triggering certain fears may be difficult to reproduce in real life [25]. The scripted nature of a virtual reality app prevents the occurrence of unexpected situations and provides patients with a safe environment where they can experience specific aspects of life, allowing them to be “emotionally present” within the virtual environment [26]. Immersive virtual reality provides an even more intense experience for the user, using a virtual reality headset or multiprojected environments to generate realistic images, sounds, and other sensations [25].

Virtual reality exposure therapy has been successful in modifying body image distortions in people with obesity, binge eating disorder, and eating disorders not otherwise specified, leading to a reduction in inappropriate behaviors related to food and social relationships [27].

Besides modifying body image distortions, virtual reality studies of eating disorders have focused on exposure to virtual food stimuli [23]. Some of these studies aimed to assess emotional reactions or craving while using virtual reality, while others had therapeutic objectives [23]. The results suggest that the participants perceived the virtual reality environment as real and that it was able to elicit emotional responses (eg, anxiety, food-related thoughts) [23]. More specifically, for binge eating disorder and bulimia nervosa, studies indicate the potential utility of virtual reality in improving motivation for change, self-esteem, and reducing binge eating and purging behaviors [20].

In obesity, besides modifying body image distortion, virtual reality studies were successful at improving self-control, mood, and weight loss [28-30]. Another area of focus was to increase physical activity using “exergaming” as a potential treatment for obesity and diabetes. Exergaming's fundamental concept is to use vigorous body activity as the input for interacting with digital game content, in the hopes of replacing the sedentary activity that is typical of traditional keyboard, game pad, and joystick inputs [31,32].

In addition to these findings, a study that investigated the perceived usability of a virtual reality app that promoted normal eating behavior suggested that clinicians have a positive attitude toward using virtual reality when treating people with an eating disorder [33]. Considering the advantages provided by this technology, virtual reality could be a useful tool to address eating disorders and obesity via exposure therapy and behavioral training.

Although interest in using virtual reality for treating eating disorders and obesity has increased over the last 2 decades, there is a lack of studies examining the effect of behavioral training via virtual reality on eating behaviors and satiety [22,23]. It has been proven that better treatment outcomes are obtained when the treatment normalizes eating behavior [34]. Similar methods may be successful in treating other eating behavior problems. Thus, a behavioral training approach via virtual reality could provide a new adjunctive tool to the existing treatment of eating disorders and obesity.

Both the method (eg, virtual reality and immersive virtual reality) [23] and aim (eg, normalizing body image and food exposure) [22,23] of virtual reality studies in eating disorders and obesity have been inconclusive, and further work is required to clarify these findings. Another reason to investigate the effectiveness of virtual reality further is that clinicians have expressed concerns that people with anorexia nervosa could substitute virtual meals for real meals to reduce feelings of hunger without ingesting any food [33].

Therefore, the main aim of the study was to determine whether eating in an immersive virtual reality environment reduces hunger among healthy adults. The secondary aim was to describe any eating behavior differences between the consumption of virtual reality meals and real-life meals.

## Methods

### Participants

General inclusion criteria were women, 18 years to 28 years of age, a BMI from 18.5 kg/m^2^ to 29 kg/m^2^, not pregnant nor breastfeeding, and normal physical activity (<1500 metabolic equivalent of task min/week of moderate-to-vigorous physical activity level from the International Physical Activity Questionnaire). Health inclusion criteria were nonsmokers, the absence of temporomandibular disorders or recent serious dental surgery, not undergoing treatments known to affect appetite (eg, some psychotropic drugs, acetazolamide), and no previous history of eating disorders. Only nonvegetarians with no aversion to the food that was to be served were allowed to participate in the study.

### Recruitment

Recruitment of participants started in October 2020 and ended in April 2022. Participants were recruited via digital meeting platforms, by contacting potential participants in person, and by hanging flyers around the university campus.

### Instruments

#### BMI

To measure BMI, weight was measured using a body composition analyzer (Tanita BC-418), and height was measured using a stadiometer (Seca 216 1814009). To collect data on eating behavior (ie, chews, bites, forkfuls) a home security camera (Mi 360°, Xiaomi) was used. To display and interact with the virtual meal, we used a virtual reality headset (HTC VIVE Pro), which consists of a head-mounted device and accompanying handheld controllers. The headset provides 110 degrees of viewing at a resolution of 1440 x 1600 pixels per eye (2880 x 1600 pixels combined) and a refresh rate of 90 Hz.

#### Food Intake

To measure food intake throughout the real meal, a digital food scale (Mandometer version 4) was used. The Mandometer is a medical device that consists of a scale that is connected to a computer; the scale measures the weight of the food on the plate at a sampling frequency of 1 Hz [35]. Before starting a meal, a plate was placed on the food scale, and the weight of the plate was subtracted from the meal. Food was then served on the plate, and the weight of the food was registered throughout the meal.

#### Subjective Data

Subjective data were collected via a tablet (Samsung Galaxy Tab A7) using an in-house developed app containing all questionnaires to ensure that participants fulfilled inclusion criteria, and it also contained 1 questionnaire to collect outcome data.

#### Inclusion Questionnaires

The short form of the International Physical Activity Questionnaire was used to evaluate participants' physical activity and to exclude those with too high moderate-to-vigorous physical activity levels [36]. The questionnaire consists of 7 questions, for example “During the last 7 days, on how many days did you do vigorous physical activities like heavy lifting, digging, aerobics, or fast bicycling?” The Health questionnaire was used to exclude individuals who were not healthy enough to participate or did not fulfill the inclusion criteria. The questionnaire consists of 25 questions about the general health of the participants (eg, “Do you often have infections like cough or sore throat?”). The Dutch Eating Behavior Questionnaire was used to assess 3 distinct eating behaviors (external, emotional, and restrained eating) [37]. The questionnaire consists of 33 questions, such as “If you have put on weight, do you eat less than you usually do?”, “Do you have the desire to eat, when you are emotionally upset?”, or “If you see others eating, do you also have the desire to eat?”

#### Outcome Questionnaire

Subjective appetite, mood, and taste of the food were rated using a meal questionnaire, which consists of 25 questions divided into 2 parts. The first part, “before meal,” consists of 6 questions about mood (eg, “Are you feeling tense right now?”) and 6 questions about appetite (eg, “How hungry do you feel?”). Mood and appetite questions were rated on 100-mm visual analog scales ranging from 0 to 100, with the verbal anchors “Not at all”=0 on the left side and “Extremely”=100 on the right side. The second part, “after meal,” consists of 6 questions about appetite (same as before the meal) and 7 questions about the taste of the food (eg, “How fatty did the food taste?”) on visual analog scales identical to that presented before the meal.

### Ethical Approval

The research protocol was approved by the Swedish Ethical Review Authority (D.nr: 2019-04249).

### Procedure

**Figure 1:**
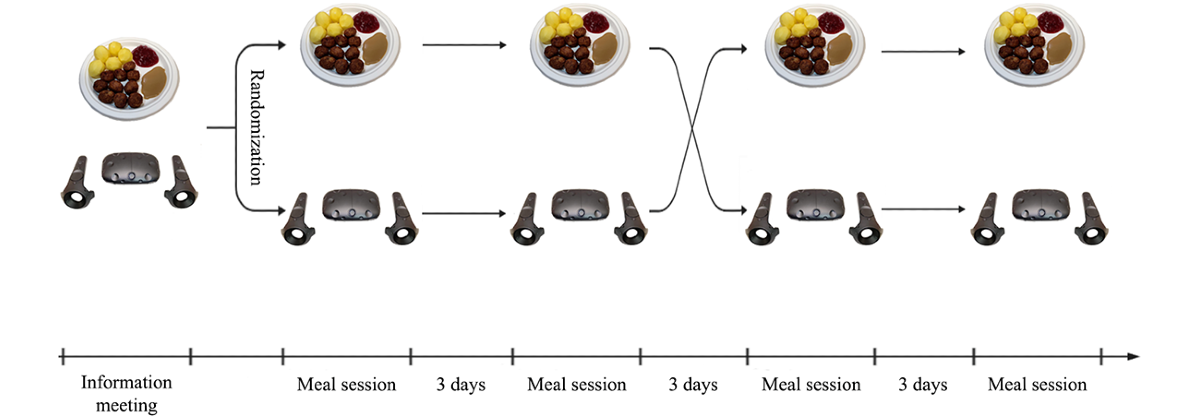
Study design.

The study consisted of 5 meetings in a randomized crossover design, in which participants acted as their own control with 2 conditions, each with 2 repeats. Respondents first attended an information meeting where they ate an introduction meal. During the remaining 4 meetings, participants were either served a virtual or a real meal in a randomized order (Figure 1). All 5 meetings, including the information meeting, were held on weekdays (Monday to Friday) during lunch hours (11 AM to 1 PM) and had a wash-out period of at least 3 days. Both the real-life and virtual reality meals were recorded using a video camera.

The day before each meeting, participants received an email with information regarding the type of meal (virtual or real) that they would have. To control for satiety, participants were reminded to eat the same breakfast the day of the meeting, at least 3 hours before, and to refrain from high-intensity physical activity 24 hours before the meeting. All the real meals were prepared by the researchers in the clinic 30 minutes to 60 minutes before the participants' arrival. All the prepared meals were kept in the oven at a temperature of 80 °C to ensure proper serving temperature. In the virtual world, the size of utensils, food containers, and food items were equivalent to the matching real-world item. Size-to-weight of food items in the virtual world was calculated, and corresponding meatballs and potatoes were purchased for the real-world meals, resulting in meatballs weighing 14 g each and potatoes weighing 60 g each.

### Information Meeting

During the information meeting, respondents were provided with detailed information about the study, data collection, handling and secrecy, and the potential risks associated with participating. Besides informing them, the purpose was to ensure their eligibility to participate in the study, familiarize them with the study procedures, and provide a baseline food intake value for the rest of the meals. Each participant signed a written consent form and proceeded to answer the Health and the International Physical Activity Questionnaires. Once the questionnaires were reviewed and approved, the participant’s height and body composition were measured.

Afterward, the introduction meal was served. In total, 1.2 kg of food was served, consisting of meatballs (400 g) and boiled potatoes (800 g). Brown sauce and lingonberry jam were placed in separate ceramic bowls on the table with a glass of water (200 mL). After the food had been served, the participant filled in the “before meal” part of the meal questionnaire. Once completed, the participant was instructed to put food on the plate that had been placed on the food scale and to eat the meal as they normally would, with no time nor intake restriction (ad libitum). Finally, instructions were given for the participant that they could add food to the plate or leave food on the plate and to refrain from using a mobile phone or other distracting elements while eating. Once the participant was finished putting food on the plate, the food scale and the video camera were turned on. After meal termination, the participant filled in the “after meal” part of the meal questionnaire.

The next step of the procedure was the virtual meal, for which the participant was taken to the virtual reality room, seated on a real chair in front of a real table, and fitted with the virtual reality equipment. The virtual reality room showed a kitchen that included a table, chair, and windows that revealed a natural scene. In the virtual reality environment, the controllers were represented by hands. The introduction meal consisted of several steps, teaching the participant how to serve themselves and consume food in the virtual reality environment [33]. A virtual tablet presented what tasks to perform to proceed to the next step. Each step ended by pressing the “next” button. In every step, a new “object” appeared on the table starting with a plate and continuing with meatballs in a pan, potatoes in a pot, brown sauce in a sauceboat, lingonberry jam, a jug of water, a glass, and cutlery [33]. After completing all previous steps, the participant could freely “cut” and “eat” the food.

### Real-life Meal

**Figure 2:**
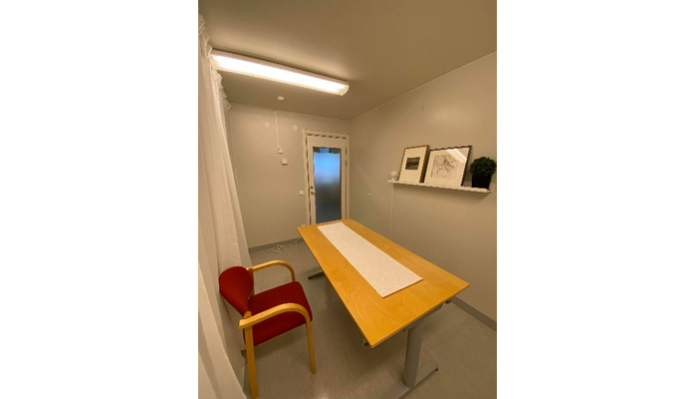
Research room.

**Figure 3:**
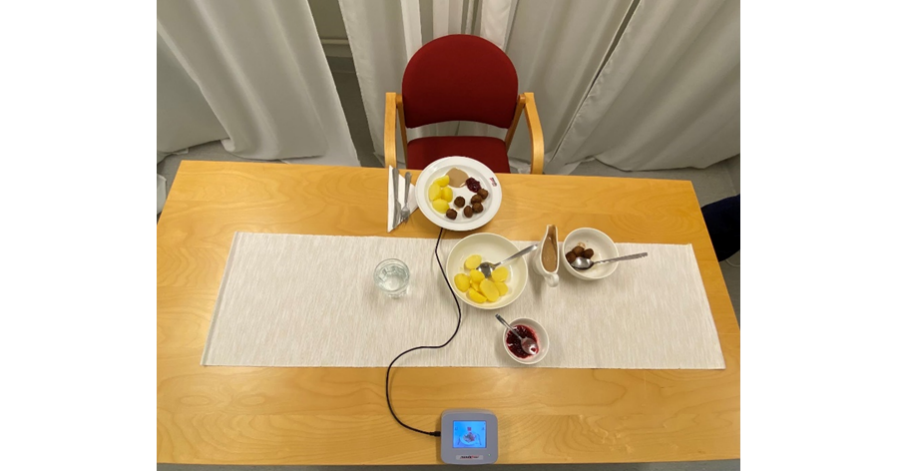
Food was served, and 50% of the portion was placed on the side.

The meeting for the real-life meals started by measuring the participant's body composition and filling in the “before meal” part of the meal questionnaire. The participant was then led to the room where they would eat. The room was designed to be as neutral as possible, with only a table and a chair (Figure 2). The meal was served on a plate that was placed on the food scale, with a glass of water alongside. The quantity of food on the plate was the same as the amount of food that the participant consumed during the introduction meal, plus an extra 50% portion on the side (Figure 3). The same instructions as in the introduction meal were provided. Once the participant was done eating, they filled in the “after meal” part of the meal questionnaire.

### Virtual Meal

**Figure 4:**
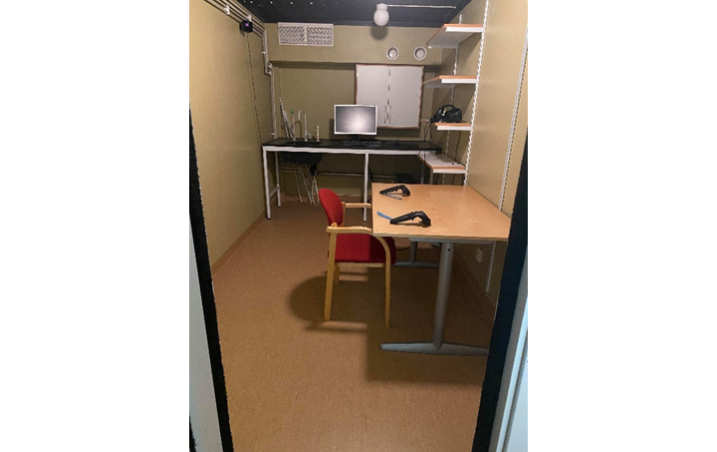
Virtual reality room.

**Figure 5:**
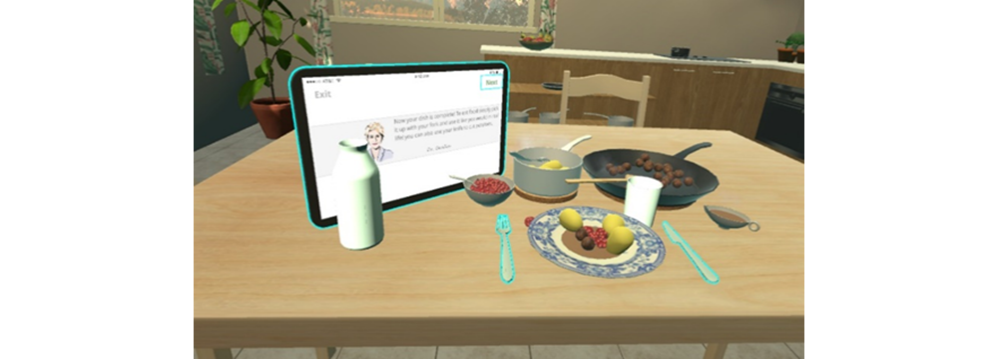
Virtual reality environment.

The meeting started by measuring the participant’s body composition. Afterward, the participant was taken to the virtual reality room (Figure 4), filled in the “before meal” part of the meal questionnaire, and was fitted with the virtual reality equipment. The meal served in virtual reality was the “same” as the real meal; the portion of the virtual meal was equivalent to the portion that was consumed during the introduction meal. There was extra food in the containers placed on the table, enabling free addition of food to the plate (Figure 5). Once the participant was ready to eat the virtual meal, the video camera and the acquisition software from the computer were initiated by the researcher. The purpose was for the meal to be eaten normally with no time restriction. Once the participant was done, the “after meal” part of the meal questionnaire was filled in.

### Data Formatting

Video recordings from both virtual and real meals were annotated using The Observer XT 16 (Noldus). The eating-related behaviors annotated were forkful, food addition, bite, and chew. Forkful was annotated as a point event when the fork touched the plate to pick up food. Addition was annotated as a state event, starting when food was added to the plate and ending when no more food was added and food ingestion started again. Bite was annotated as a point event when the mouth was closed around the fork. The virtual food did not require bites, as the food was “eaten” by putting it close to the headset. Chew was annotated as a point event when teeth occlusion and activation of masseter and temporalis muscles occurred.

### Statistical Analysis

Statistical analyses were carried out using R software version 4.2.1 [38]. Since participants conducted 2 meals for each eating condition (2 real and 2 virtual meals), the average of every measured outcome (eg, food intake, hunger) was calculated. Thus, for each eating condition, 1 value (the mean) was created and was used in the analysis. For example, the food intake of the virtual reality meal refers to the mean food intake from the first and the second virtual reality meals.

The Shapiro-Wilk test and visual inspection of a Q-Q plot were performed to ensure normal distribution of the data. For the parameters that were not normally distributed, a Wilcoxon signed rank test and a paired Student *t* test were performed. Since the interpretation (ie, rejection or nonrejection of the null hypothesis) to both tests were identical for all investigated parameters, only *t* tests are presented in the results. The relevant parameters tested were hunger before and after the virtual and real meals, food intake, meal duration, eating rate, forkfuls, and additions, between the real and virtual meals. A subgroup was created from the participants who took chews and bites in the virtual meals. Additional *t* tests were performed for this subgroup, comparing chews and bites in the virtual meal to the real meal. Last, the same test was performed for all participants to evaluate the effect on hunger and meal duration between the first and second meals in both eating conditions. Pearson product-moment correlation was used to examine the association between hunger after the meal and meal duration and chews, as well as the correlation between meal duration and chews and food intake. Caution is required when interpreting all correlation analyses including chews from the virtual reality condition as a parameter, since in more than one-half of the measurements, the number of chews was 0. A significance threshold of *P*≤.05 for all statistical tests was used. Unless otherwise specified, values are expressed as mean (SD). For the power calculation, a sample size of 20 was found to be sufficient for the study, using the effect size between real meal conditions of previous studies, an α cutoff of .05, and power of 0.80.

## Results

### Participants

Of 24 participants, 20 finished all parts of the study (Table 1); 2 participants dropped out due to scheduling difficulties, 1 participant was excluded due to corrupt data, and 1 participant was excluded due to suspected conformity bias, based on low food intake. No participant was excluded based on questionnaire responses.

**Table 1:** Demographic data of the participants (n=20).

Demographic characteristics	Results, mean (SD)
Age (years)	23.9 (2.5)
Weight (kg)	61.2 (9.6)
Height (cm)	165.3 (7.4)
BMI (kg/m^2^)	22.4 (2.8)
Body fat (%)	27.5 (5.1)

### Hunger Before and After Meals

For the virtual meal, participants rated their hunger before and after the meal at 66.9 and 59.9 (out of 100), respectively, resulting in no significant difference in hunger before and after the virtual meal (*P*=.10). For the real meal, participants rated their hunger before and after the meal at 68.6 and 6.9, respectively, resulting in a significant reduction in hunger after the real meal (mean difference=61.8, *P*<.001). One participant had a reduction in hunger similar in size to what was expected for a real meal as a result of eating a virtual meal, with a rated reduction of 59.

There was no significant difference in hunger before the meal between the virtual and real meals (*P*=.79), but hunger was significantly higher in the virtual condition after the meal (mean difference=–53.1, *P*<.001).

### Eating Behavior Characteristics

There was no significant difference in “food” intake between the virtual and real meals (*P*=.07). Meal duration was significantly shorter with the virtual meal (mean difference=–5.4, *P*<.001), which led to a higher eating rate (mean difference=82.9, *P*<.001) for the virtual meal. When the chewing time (burst interval) was removed from the virtual and real meals, there was no significant difference in meal duration between the virtual and real meals (*P*=.06). The number of forkfuls and number of additions were significantly lower with the virtual meal than with the real meal (mean difference=–13.0, *P*<.001 and mean difference=–2.0, *P*=.005, respectively). The results are presented in Table 2.

**Table 2:** Eating behavior characteristics from the virtual and real meals (n=20).

Eating behavior	Virtual meal, mean (SD)	Real meal, mean (SD)	*P* value
Food intake (g)	375.6 (140.9)	338.9 (108.3)	.07
Meal duration (min)	3.6 (1.7)	9.0 (3.0)	<.001
Meal duration without chews (min)	2.8 (1.3)	2.4 (1.2)	.06
Eating rate (g/min)	124.0 (52.9)	41.1 (16.9)	<.001
Forkfuls (n)	25.9 (9.8)	38.9 (14.8)	.002
Additions (n)	0.6 (0.8)	2.5 (2.9)	.005
Bites (n)	10.0 (11.7)	40.0 (15.8)	<.001
Chews (n)	28.9 (68.0)	419.0 (162.9)	<.001

### Chews and Bites

For participants who took bites and chews in the virtual meal, the numbers were significantly lower compared with the real meal (n=14, mean difference=–28.1, *P*<.001 and n=6, mean difference=–387.4, *P*=.004, respectively). The results are presented in Table 3.

**Table 3:** Mean values from participants who took bites (n=14) and chews (n=6) in the virtual meal.

Eating behavior	Responding (n=20), n (%)^a^	Virtual meal, mean (SD)	Real meal, mean (SD)	*P* value
Bites	14 (70)	15.4 (11.2)	42.4 (16.1)	<.001
Chews	6 (30)	82.4 (97.5)	469.8 (193.0)	<.001

^a^Number included in the analysis, based on the participants who took bites or chews in the virtual meal.

### Familiarization Effects

There was a significant reduction in meal duration from the first virtual meal to the second virtual meal (mean difference=0.4, *P*=.03) but no significant change in meal duration between the first real meal and second real meal (*P*=.07). There was no significant difference in hunger before the meal between the first virtual meal and second virtual meal and between the first real meal and second real meal (*P*=.40 and *P*=.22, respectively).

### Association Between Hunger and Chews and Meal Duration

In both the virtual and real meals, a low correlation was found between hunger after the meal and number of chews (r=–0.10, *P*=.68 and r=–0.18, *P*=.44, respectively). A low correlation was also found between hunger after the meal and meal duration in both the virtual and real meals (r=–0.12, *P*=.60 and r=–0.12, *P*=.62, respectively).

### Association Between Meal Duration and Chews and Food Intake

In both the virtual and real meals, a low correlation was found between meal duration and food intake (r=0.22, *P*=.35 and r=0.14, *P*=.56, respectively). A high correlation was found between meal duration and chews in the virtual and real meals (r=0.69, *P*<.001 and r=0.75, *P*<.001, respectively).

## Discussion

### Principal Findings

To our knowledge, this is the first study to investigate the effect of a virtual meal on hunger and to explore the differences in eating behavior between real and virtual meals. The findings of this study indicate that eating a virtual meal does not affect hunger in healthy women. The results demonstrated that participants consumed approximately the same amount of real and virtual “food,” but the virtual meal was significantly shorter, resulting in a higher eating rate. Participants also had lower numbers of chews, bites, forkfuls, and additions in the virtual meal.

### Comparison With Prior Work

It is expected for hunger to decline throughout a real meal, which was the case for the real meal condition. Meanwhile, eating a virtual meal did not significantly reduce hunger. The nonsignificant difference in hunger before and after a virtual meal suggests that such meals can be used for people with anorexia nervosa without the risk of having them replacing real meals with virtual meals. This finding is in contrast with a study in which participants were found to decrease their consumption of a particular food (eg, cheese) by repeatedly imagining eating it [39]. Since this study was conducted with healthy women, care will be required when implementing this method for eating disorders or obesity.

The extent to which a virtual meal represents realistic eating conditions was also examined, although the aim of the virtual reality app was not to mimic real-life eating behavior. Interestingly, even though the meal duration of the virtual meal was shorter than that of the real meal, the amount of “food” consumed was approximately the same. The reason for this phenomenon was that most of the participants did not chew during the virtual meal, resulting in an approximately 3 times higher eating rate compared with the real meal. These data support previous research, which showed that eating rate was inversely related to the number of chews [40]. Changing the virtual reality protocol to elicit a more realistic eating behavior or providing virtual reality eating training may result in a satiety response. However, findings suggest that this approach would not be beneficial as an intervention for obesity or bulimia nervosa.

A serendipitous finding of the study was that, although it was not required nor requested, participants took bites and chews during the virtual meals, indicating that the virtual reality environment was perceived on some level as being real. The display of eating behaviors similar to real life is in line with the findings that cue exposure in virtual environments is effective for inducing food craving in healthy control participants [41].

The familiarization effect observed in the virtual meals suggests more meals are required to reach a stable behavior since several participants experienced difficulties. Examples of those difficulties were placing the food in their mouth and grabbing or serving the food on their plate. People are less accustomed to eating virtual meals than real meals, and that conclusion is also supported by a study that examined the impact of the environment on eating behavior using virtual reality [42].

Based on previous findings, we expected longer meals (ie, longer exposure to food “images”) [43] and more chews (ie, more oral processing) [44] to be negatively associated with hunger. However, the correlation was low in both cases. These correlations should be interpreted with care, since they were not part of the initial hypothesis (we did not know participants would chew virtual food) and the fact that more than one-half of the participants did not chew in the virtual meal.

### Strengths and Limitations

The main strength of this study was the use of immersive virtual reality equipment, as this technology increases the feeling of being present in the environment [20]. Other strengths were that many confounding covariates were controlled through the deployment of a random crossover design [45], narrow inclusion criteria, a strict study protocol [42], a familiarization meal, and objective data collection methods. For the introduction meal, a larger portion (1.2 kg of food) than what is typically ingested was offered to the participants to make them realize they did not have to consume everything. For the coming meals, they were provided with the same portion size as they ate during the introduction meal with extra food on the side. The artificial manipulation of portion size ensured similar meal sizes, making hunger, eating rate, and other eating behavior parameters comparable [46].

The main limitation of the study was the novelty of immersive virtual reality. Participants unused to immersive virtual reality require practice and concentration to understand the “game” mechanics [42]. It was observed that, in the introduction meal, several participants experienced difficulties handling the food (eg, placing the food in their mouth and grabbing or serving the food on their plate). Some participants expressed surprise or amusement during their encounter with virtual reality equipment (especially the first time), which may have influenced the results. Another limitation was that the virtual reality app required food on the plate and in containers to start, which resulted in alterations to the real meal protocol as well. Ideally, participants would have only received food on the plate for each session.

Other limitations include a lack of effect size measurements from previous virtual reality meals for the power analysis and potential low generalizability to people with eating disorders or obesity, because the study enrolled only healthy women. Moreover, the recruitment location (university campus) may have resulted in a convenience sample, in regard to education, socioeconomic status, technological literacy, and availability (workdays from 11 AM to 1 PM). In addition, the subjective methods used to quantify hunger are vulnerable to demand bias, in which the participants provide responses that they believe the researchers are looking for [43]. However, there are no reliable biological markers to evaluate appetite [47].

### Future Perspectives

Further investigation is necessary to confirm the safety of virtual reality for patients, as they may respond differently than healthy control participants. Future studies should aim to include other demographic groups (eg, men, children, vegans, and other groups prone to eating disorders or obesity) to evaluate the generalizability of the findings. The effect of a virtual meal should also be investigated in different settings (eg, at home, at a restaurant, lunch with friends) and with a variety of foods. Moreover, future studies should provide additional virtual meals to reduce the familiarization effect observed in this study. Looking to the future, further attempts at incorporating chewing in the virtual meal (eg, with chewing gum to mimic food mastication) and using more advanced equipment that allows the involvement of all the senses (eg, sounds and olfactory information) might prove beneficial in evoking physiological responses similar to those of real meals.

### Conclusions

This study investigated the effect of virtual reality on hunger. Eating in an immersive virtual reality environment has no significant effects on hunger in healthy individuals, suggesting that virtual reality can be used for patients (eg, with anorexia nervosa) without the risk of having them replace real meals with virtual meals. Due to the lack of chewing, the virtual meal was consumed in less time, while the food intake was approximately the same as in the real meal. Surprisingly, some participants took bites and chews in the virtual meal, although they were not instructed to nor was it required to “eat” the food.

## Appendix

Multimedia Appendix 1 CONSORT-EHEALTH checklist (V 1.6.1).
